# National Education

**Published:** 1901-09-21

**Authors:** 


### The Hospital

A Journal of

### The Medical Sciences and Hospital Administration.

Vol. XXX.—No. 782.] Edited by Sir Henry Burdett, K.C.B., and Solomon C. Smith, M.D., M.R.C.P. [September 21, 1901.

### NATIONAL EDUCATION.

It can scarcely be questioned that the formation of an educational section of the British Association for the advancement of science, and especially its formation under the Presidency of the minister most immediately responsible to Parliament on educational questions, is an event full of good omen for the future. Education, in this country, has hitherto been frankly empirical. The best schoolmasters have been men gifted with certain intuitions concerning the management of the young; and their methods, although frequently described with more or less inaccuracy from the points of view of different observers, have never been reduced to any general or intelligible system by themselves. Their business, more especially in relation to the children of the upper and middle classes, has, in the forcible and simple language of Dr. Dukes, been the only one for which men intending to pursue it were not trained. It is more than likely that even the most successful among them, the teachers or disciplinarians whose names have become household words, would either have been unable to give any intelligible explanation of their own success, or would have attributed it to conditions of which it was entirely independent. What was to be the mental development of any particular boy or girl, how far his or her capacities were to be developed or repressed by environment or teaching, has depended upon the cast of a die. The idea of adapting studies to the intellectual or moral requirement of the taught has either never been entertained at all or has been dismissed as chimerical. The conception that observation, reflection, and memory, with all their consequences and fruits, are mere functions of brain tissue, dependent for their character upon the physical qualities of that tissue, and capable of calculable modification by all manner of external conditions, has never been brought home to the hundreds of worthy people who have chattered about "mind," and who have fancied that their chatter was an expression of ideas. Physiology, which must form the basis of every rational system of teaching, has, in the enormous majority of cases, been an absolutely sealed book to the teacher, with this one result, among many others, that facts of the highest possible importance to his calling have been daily passing unperceived before his eyes. Another result has been an almost complete absence of progress in educational methods. The successful man has increased the reputation of the school over which he presided, only that it might decline again under the control of his successor. There has been no "science," no enlightened application of established principles to the attainment of definite ends. Great men have been produced, some of them perhaps as consequences of the training they had received; more, in all probability, in spite of it. As a rule, the chief object aimed at has been to impart knowledge, leaving the learner to discover its uses and applications. It has been as if a *maître d'armes* were to give his pupil a rapier, and to tell him to find out for himself its uses as a weapon of offence or defence. The not unnatural result, perhaps even the inevitable result, has been that on the continent of Europe, where education, like other arts, has been stimulated by the force of eager competition in the walks of industry and commerce, methods have at least been so far improved as to provide large numbers of young men and women who are better trained than is customary among ourselves to play their parts in the battles of life.

In the meanwhile, the operations of the growing brain have been studied, to some extent in this country, and still more largely in the United States, by observers bent upon ascertaining the natural processes by which these operations are gradually improved and perfected, and the methods by which it may be possible not only to assist and develop the weak, but also to call forth all the strength of the strong. Close observation and careful study have been bestowed upon the gradual formation of paths of association between related ideas, or between ideas and the actions which they are calculated to produce. Curiosity, as the foundation of all knowledge, is being recognised as a faculty to be encouraged, and the wants of which should be sedulously supplied. The period at which children were told "not to ask questions," has, it may be hoped, at least with regard to a large proportion of the middle and lower middle classes, passed away for ever; even although more than vestiges of its existence may still be discovered in the nurseries of the wealthy. The origins of reform have so far shown themselves chiefly in comparatively humble localities, in the work of Kinder Garten schools, in lectures given in suburban institutes, and so forth; but sufficient evidence of improvement is even now forthcoming to justify considerable hopefulness that valuable human material will be less likely to run to waste in the future than it has been in the past. The step taken by the British Association is the first recognition of the existence of a science of education as distinguished from an art or a calling; it is the first peal of the trumpets with which the reformers are commencing their march around the walls of the scholastic Jericho which it is their mission to capture and to subdue. It is manifest that the work of the trained observer of the mental and bodily operations of the young must go hand in hand with that of the physiologist who seeks to penetrate the secrets of nervous function and of nervous activity, and that the results of each must be corrected or supplemented by those of the other. In the meanwhile it is much that the fetishes chiefly worshipped by schoolmasters have been scornfully turned inside out by the Vice-president of the Board of Education, and that, for the future, the seekers after sounder principles and better methods will have their places on the same platforms with the leading exponents of the most advanced science of the day.

